# The Glomerular Filtration Barrier: Components and Crosstalk

**DOI:** 10.1155/2012/749010

**Published:** 2012-08-14

**Authors:** Madhav C. Menon, Peter Y. Chuang, Cijiang John He

**Affiliations:** ^1^Division of Nephrology, Mount Sinai School of Medicine, New York, NY 10029, USA; ^2^Division of Nephrology, James J. Peters VA Medical Center, Bronx, NY 10468, USA

## Abstract

The glomerular filtration barrier is a highly specialized blood filtration interface that displays a high conductance to small and midsized solutes in plasma but retains relative impermeability to macromolecules. Its integrity is maintained by physicochemical and signalling interplay among its three core constituents—the glomerular endothelial cell, the basement membrane and visceral epithelial cell (podocyte). Understanding the pathomechanisms of inherited and acquired human diseases as well as experimental injury models of this barrier have helped to unravel this interdependence. Key among the consequences of interference with the integrity of the glomerular filtration barrier is the appearance of significant amounts of proteins in the urine. Proteinuria correlates with kidney disease progression and cardiovascular mortality. With specific reference to proteinuria in human and animal disease phenotypes, the following review explores the roles of the endothelial cell, glomerular basement membrane, and the podocyte and attempts to highlight examples of essential crosstalk within this barrier.

## 1. Proteinuria

Urinary protein excretion in the normal adult humans is less than 150 mg/day. Persistent protein excretion greater than this merits further evaluation. Proteinuria is strongly associated with progression of kidney disease [1]. Furthermore, proteinuria has proven to be an independent risk factor for all-cause and cardiovascular mortality [2]. Proteinuria can be glomerular resulting from an impairment of the glomerular filtration apparatus, tubular from diminished tubular resorption of low-molecular-weight proteins, and overflow—where the resorptive capacity is overwhelmed by large loads of filtered proteins [3, 4]. Although some studies have suggested that the filtration barrier is more permeable to albumin than previously reported [5, 6] thus attributing a more significant role for the proximal tubules in determining the magnitude of proteinuria, more recent studies have disputed those findings and corroborated classical measurements of the glomerular sieving coefficient derived from micropuncture experiments [7, 8]. For the purposes of this paper, we will restrict our discussion to molecular and structural pathomechanisms of glomerular proteinuria. 

## 2. Glomerular Filtration Slit Diaphragm: A Multicomponent Apparatus

**Figure 1:**
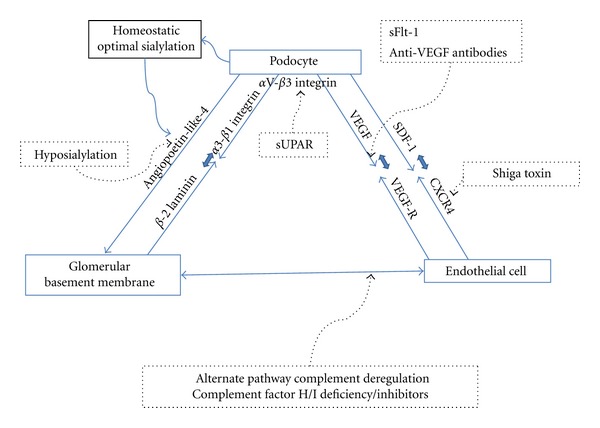
Components of the Glomerular filtration barrier with examples of crosstalk. This figure summarizes important signaling interactions between the 3 key components of the GFB and their putative involvement in human disease models (dotted arrows). Podocyte *α*3-*β*1 integrin interacts with GBM *β*-2 laminin; hyposialylated-podocyte-secreted Angp-4 may have an effect on the GBM and the endothelial cell in MCD models. sUPAR, a potential circulating “serum factor,” affects podocyte *α*V-*β*3 integrin in primary FSGS. Podocyte-endothelial cell interactions are affected by VEGF/VEGF-R blocking agents in preecclampsia while SDF-1-CXCR4 pathway is perturbed by Shiga toxins in HUS. Alternate pathway complement dysregulation and consequent endothelial components are central to MPGN and atypical HUS.

The filtration apparatus is complex; its integrity is maintained by an interplay of all participating cell types and constituents [9]. The glomerular filtration barrier (GFB) is freely permeable to water, small- and midsized solutes in plasma, yet maintains considerable size and charge selectivity for proteins and larger molecules. This barrier has three major components: the fenestrated endothelial cell, the glomerular basement membrane (GBM), and the podocyte with their “slit diaphragms”. In all pathologic glomerular proteinuria, there is increased filtration of macromolecules (typified by albumin) across this barrier. Injury to this apparatus can be pathogenetically classified as resulting from inherited and acquired causes (Table 1). Inherited human diseases presenting with defects in the GFB have been shown to involve abnormalities of proteins important for the maintenance of podocyte cytoskeleton [10], glomerular basement membrane integrity [11], and glomerular endothelial function [12]. Possibly owing to its specialized structure and abundant blood flow, the glomerulus is uniquely susceptible to both immunological and hemodynamic injury [13]. In addition to discussing the roles individual components in the GFB play in glomerular function in health and disease, we will attempt to highlight the accumulating evidence for significant interdependence and cross-talk within this unit (Figure 1).

## 3. Roles of the Podocyte

Podocytes are terminally differentiated, highly specialized epithelial cells of mesenchymal origin located on the urinary aspect of the GFB. The surface of podocytes is covered by anionic glycocalyx, constituted mainly by podocalyxin [14]. Podocytes are characterized by their foot processes, a network of interdigitating cellular extensions (primary, secondary, and tertiary), which support the glomerular capillary loop from the visceral aspect of the Bowman's space and interact at specialized cell-to-cell junctions called slit diaphragms [9]. Slit diaphragms contain proteins that are typically found in tight junctions (i.e., zona occludens-1 [15]) as well as adherens junctions (catenins, P-cadherin [16]). The critical roles of the integral proteins of the slit diaphragm and foot processes have been strongly implied by their association with familial nephrotic syndrome in humans (Table 1) and the renal phenotypes observed in knockout animals. In vitro studies on podocytes have been limited by the inability to reproduce the in vivo podocyte cytoskeletal phenotype in cell culture. Nephrin is a transmembrane protein member of the immunoglobulin superfamily protein found in the slit diaphragm [17]. Its interaction with nephrin (homologous) from an adjacent podocyte or neph-1 (heterologous) is hypothesized to be the “pore” of the slit diaphragm [18]. The intracellular domain of nephrin interacts with podocin and other regulatory proteins to facilitate actin polymerization [18, 19]. The polymerization and specific arrangement of actin filaments is the key to foot process architecture. This is supported by the identification of hereditary nephrotic syndrome with foot process abnormalities resulting from single-gene mutations in a number of actin-binding proteins involved in actin polymerization such as *α*-actinin-4 and inverted formin 2 [20, 21].

 The podocyte slit diaphragm is the final barrier in the GFB to filtration of macromolecules. Distortion of foot process architecture resulting in foot process effacement (FPE) or flattening is observed in the majority of instances where glomerular proteinuria is present with a few notable exceptions. These exceptions [23, 24] argue that foot process effacement is neither necessary nor sufficient to induce proteinuria. The best example is preecclampsia where nephrotic proteinuria is unaccompanied or variably accompanied by effacement [25]. The reported evidence for FPE without proteinuria is less profuse. Glomerular epithelial protein 1 (GLEPP1) is a receptor tyrosine kinase located on the apical surface of podocytes. Homozygous GLEPP1-knockout mice developed abnormally shaped podocytes with FPE but did not develop proteinuria [26]. Additionally, even in diseases where effacement is classically encountered, its quantification by morphometry does not seem to correlate with the degree of proteinuria [27]. FPE as a reversible event, induced by infusion of polycations and reversed by polyanions was classically demonstrated in rats [28]. This concept of FPE as an ongoing process reversibly initiated by podocytes in response to injurious stimuli is reemerging from multiple observations [29, 30]. The best evidence for near complete reversibility of FPE comes from minimal change disease (MCD) nephrotic syndrome in children and adults upon treatment [31]. FPE in FSGS on the other hand seems to represent a more irreversible event [10]. These could imply that FPE occurs as a consequence of injury to podocytes. It may also suggest that FPE in different diseases though morphologically identical may represent different ultrastructural and cytoskeletal entities. Furthermore, FPE appears to require active actin filament reorganization consequent to intracellular signals and may not merely be a passive response [32]. Deficiency of focal adhesion kinase (FAK), a cytoplasmic tyrosine kinase essential in connecting the actin cytoskeleton with the foot process anchor on the GBM, appears to protect podocytes from developing FPE in response to LPS in podocyte-specific FAK-knockout mice [22]. Upregulation in podocytes of cathepsin L, a protein associated with epithelial cell mobility has also been observed to correlate with FPE [33]. This finding may suggest the adoption of a cytoskeletal phenotype by podocytes undergoing FPE that favors motility, often as a response to injury [33]. 

 The interaction between *α*3-*β*1 integrin and *β*-2 laminin, and its link to the podocyte actin cytoskeleton is an important part of the podocyte-GBM interaction. Anti-Fx-1A antibody that causes Heymann's nephritis in rats recognizes *α*3-*β*1 integrin and causes podocyte detachment [34]. Activation of *α*3-*β*1 integrin results in recruitment of a kinase, integrin-linked kinase (ILK). When ILK is deleted in a podocyte-specific manner, mice developed proteinuria with foot process effacement and glomerulosclerosis [35]. On the other hand, blocking TGF-*β*1 or adriamycin-induced activation of ILK using a highly selective small molecule inhibitor preserved podocyte phenotypes and ameliorated albuminuria [36]. Together, these findings suggest that the regulation of *α*3-*β*1 integrin/ILK may be important in optimal podocyte and GFB function. Urokinase-type plasminogen activator receptor (uPAR) is a glycosyl-phosphatidylinositol- (GPI-) anchored protein that is a proteinase receptor for urokinase, together forming a part of the machinery needed for cells to breakdown extracellular matrix proteins and migrate. uPAR has also been shown to be involved in nonproteolytic pathways, mainly through its ability to form signaling complexes with other transmembrane proteins including integrins [37, 38]. Among these is *α*V-*β*3 integrin in podocytes at the sites of focal adhesions [38, 39]. UPAR knockout mice appeared to be protected from FPE and proteinuria induced by LPS. Induction of uPAR signaling in podocytes lead to FPE and proteinuria via an *α*V-*β*3 integrin-dependent mechanism [39]. More recently, circulating soluble uPAR (suPAR) was found to be elevated in 66% of patients with primary FSGS and high levels appeared to confer a strong risk of recurrence of FSGS after transplant. Furthermore, high suPAR levels in mice were shown to induce FPE and proteinuria via an integrin-dependent mechanism [40]. 

## 4. Role of the GBM

Studies using metabolic labeling (experimental argyrosis) have demonstrated that GBM synthesis requires contributions from podocytes and endothelial cells, with mesangial cells playing a role in turnover [41]. The structure of the GBM has been well-characterized both morphologically and from a molecular perspective [42]. Two heterotrimeric proteins, type IV collagen (COL4) and laminin, along with sulfated proteoglycans are the preeminent constituents of the GBM. In homeostasis, *β*2-laminin principally constitutes the GBM whereas tubular basement membranes have *β*1-laminin chains [43]. Similarly, GBM COL4 is enriched in *α*-3, 4, and 5 chains whereas most other basal laminae constitute of *α*-1 and 2 chains. In the embryonic kidney *α*-1, 2 chains are still encountered in the GBM but are later completely replaced. Deficiency of COL4 *α*-5 gives rise to Alports syndrome with the most severe X-linked variant showing extensive GBM lamellations, fragmentations, and progressive glomerulosclerosis [11]. Proteinuria, though encountered is not a prominent pathogenic feature of Alport's syndrome. Autosomally inherited COL4 *α*-3 and *α*-4 chain mutations may give rise to recurrent hematuria and show only thin basement membranes on biopsy—akin to thin basement membrane disease. In mice, mutations in *α*-3 chains leads to absence of *α*-3/4/5 chains likely because heterotrimer formation with *α*-4/*α*-5 chains is disrupted. These mice develop GBM splitting, thinning, and proteinuria with podocyte FPE [44]. *β*2-laminin in the GBM interacts with and binds to *α*3-*β*1 integrin of the basal podocyte membrane as discussed above and in turn, these integrin heterodimers are linked to the podocyte actin cytoskeleton [33, 34]. *β*2-Laminin deficiency in humans leads to familial nephrotic syndrome with ocular abnormalities—Pierson syndrome [45]. Interestingly, mutant mice with *β*2-Laminin deficiency upregulate *β*1-Laminin giving the appearance of a structurally normal GBM but develop proteinuria with foot process fusion [46]. The high specificity of this interaction highlights the role of the GBM in maintaining GFB integrity and podocyte ultrastructure.

 Studies using variedly cationic ferritin first demonstrated the anionic nature of the GBM [47]. The anionic charge is mostly conferred by a lattice-like network formed by the sulfated glycosaminoglycan moieties of constituent proteoglycans [48]. Based on the increased permeability of the GFB to ferritin after heparanase treatment, the GBM was initially thought to be the source of charge selectivity in the GFB [49]. This hypothesis has been called into question more recently. Spherical Ficoll/Ficoll sulfate particles of differing negative charge did not show any charge-dependent variation in filtration fraction in isolated GBM preparations [50]. The authors preferred Ficoll as its shape and structure are less alterable with changes in charge, which contrasted with previous studies which had used radio-labelled peptides (albumin) or dextran [51, 52]. Since podocytes are the site of synthesis of heparan sulfate (HS), Chen and colleagues used a Cre-loxP strategy to target the podocyte enzyme, HS polymerase (EXT1) [53]. Mice in whom Cre recombinase expression driven by a podocyte-specific, podocin promoter were crossbred with animals in whom exon 1 of EXT1 was floxed. The resultant mice had dramatically reduced (though not absent) HS staining in their GBM. However, in spite of a demonstrable loss of GBM charge by polyethyleneimine staining, they developed only mild albuminuria and glomerulomegaly at 8 months. There was also no statistically significant difference in body weights compared to controls [53]. Though this model does not take into account endothelial or mesangial synthesis of HS, it does suggest that GBM charge may not be central to GFB permselectivity.

 An illustration of the podocyte-GBM interdependence in the GFB has emerged from studies in NS models. Angiopoetin-like-4 (Angp4) is a podocyte-secreted glycoprotein whose transcript levels were observed to be upregulated in nephrotoxic-serum nephritis models. Clement et al. proceeded to generate transgenic podocin-Angp4 rats and mice with podocyte-specific upregulation of Angp4. These mice developed mild proteinuria with effacement of foot-processes. Homozygous rats, however, showed significant and selective albuminuria (100–500-fold, 90% albumin) with diffuse foot-process effacement analogous to human MCD. In puromycin nephropathy models of glomerular injury in rats, Angp4 was upregulated in podocytes. Further, glucocorticoids appeared to downregulate Angp4 and improve clinical parameters. Most interestingly in both mice and rats, Angp4 overproduction was associated with demonstrable loss of GBM charge. In human MCD patients, overexpression of Angp4 was detectable by immunohistochemistry. Hyposialylation of Angp4 residues was prominent and the disease showed improvement after feeding affected animals with a precursor of sialic acid to normalize sialylation. A parallel transgenic rat with adipocyte-specific Angp4 overproduction had no renal phenotype, excluding an effect from circulating Angp4 levels. Based on these findings, the authors hypothesize that podocyte-specific overproduction (with consequent hyposialylation) of Angp4 in response to inflammatory stimuli could progressively accumulate and interfere with GBM-to-podocyte signals causing foot-process effacement and proteinuria. The accumulation of Angp4 in the GBM may also reach the endothelial cells and affect signals to podocytes from them. Since mice which developed relatively mild disease had a similar degree of loss of GBM charge as rats, loss of anionic charge is unlikely to be the primary mechanism in this MCD model [54].

## 5. Glomerular Endothelial Cell

Due to the presence of overt fenestrations (50–100 nm size; ~20% of surface area), the glomerular endothelium was discounted early as the site of permselectivity in the GFB. Newer studies have refocused attention on the endothelium and its coating glycocalyx layer composed principally of proteoglycans [55–58]. Intralipid injection studies show this glycocalyx layer to extend to around 200 nm into the capillary lumen. Seminal work involving specialized perfusion and fixation techniques demonstrated the glycocalyx “plugs” that appear to cover these fenestrae [56]. Disruption of this glycocalyx layer by hyaluronidase and adriamycin has been shown to induce proteinuria [57]. Recently, arterial hypertonic saline infusion in rat kidneys with resultant displacement of the noncovalently bound particles of this layer was shown to increase filtration of albumin 12-fold [58]. 

 Examples of primary endothelial injury inducing damage to the GFB have been better elucidated within only the last decade. Endothelial cell activation is recognized as a component of many immune-mediated glomerular diseases [55]. From human disease phenotypes, the glomerular endothelium has appeared to be particularly susceptible to complement-mediated injury. In homeostasis, in spite of a low-grade autologous activation of both alternate and classical pathways of complement, it is protected by both soluble and membrane-bound regulators of complement [59]. Acquired deficiency or inactivating mutations of these soluble regulators, notably factor H (CFH), have been associated with a spectrum of glomerular disease, from Dense-deposit disease at one end to atypical hemolytic-uremic syndrome at the other [12]. CFH is a 150 KD plasma protein containing 20 homologous repeats (Complement-control protein domains). While the N-terminal of CFH appears to house the C3b binding site, the C-terminal is responsible for endothelial interaction. N-terminal mutations in CFH and antibodies targeting this moiety appear to induce a Dense-deposit disease phenotype while C-terminal mutations and antibodies cause the development of HUS phenotype [12, 59–62]. Moreover, membrane cofactor protein (MCP; CD46) is a widely expressed transmembrane complement regulator, whose deficiency on the surface of endothelial cells also causes HUS [63]. This again brings forth the carefully regulated role of the endothelium essential to maintaining the GFB in homeostasis.

 The essential cooperation within the components of the GFB has been highlighted by the role of vascular endothelial growth factor (VEGF). VEGF-A is secreted by podocytes and localizes to the cell membrane and foot processes [64, 65]. In cultured primary and immortalized human podocytes, Foster et al. observed autocrine effects of VEGF on calcium homeostasis, cell survival, and differentiation [64]. However, podocyte-specific, VEGF-receptor-2 (VEGFR-2-) knockout mice have normal glomerular development and function whereas postnatal whole body VEGFR-2 deletion leads to marked glomerular endothelial cell and microvasculature abnormalities with proteinuria [66]. Thus, greater significance appears to be attached to the paracrine effects it exerts on endothelial cells, diffusing against the direction of filtration. VEGF-knockout mice die during early embryogenesis itself as a result of a failure of vascular formation, even prior to nephrogenesis [65]. Podocyte-specific knockout of VEGF caused hydrops and renal failure with glomeruli showing markedly diminished endothelial cell migration. Additionally, mesangial cells were absent suggesting glomerular VEGF-A is required for mesangial cell migration. Glomerular injury, in the form of endothelial swelling (endotheliosis) and proteinuria, was evident in heterozygotes implying VEGF-A has a dose-dependent role [67]. Furthermore, podocytes in these mice showed evidence of dedifferentiation. In adult glomeruli, VEGF-A also appears to be essential for the maintenance of the fenestrae [65, 67].

The significance of this VEGF-mediated interaction in glomerular disease has been demonstrable in the glomerular lesion accompanying preecclampsia, a syndrome of proteinuria, hypertension and adverse fetomaternal outcomes [25]. Soluble fms-like tyrosine Kinase, a secreted form of VEGF-receptor (sflt-1 or VEGF-R1) competitively inhibits the binding of VEGF to the membrane-bound form of the receptor. It is overexpressed in pre-ecclamptic placental tissue and increased in the serum of these patients. Serum sflt-1 levels decline after delivery [25]. Typically, the glomeruli in affected patients show endotheliosis (“blood-less” glomeruli), subendothelial fibrin deposition with relative preservation of podocyte foot processes. Two elegant experimental models have shown the crucial role of podocyte-secreted VEGF-antagonism in the genesis of these glomerular lesions. Maynard et al. injected pregnant rats (approximating second trimester in human gestation) with adenovirus encoding sFlt-1 to mimic systemic sFlt-1 overproduction. These rats developed glomerular endotheliosis and exhibited hypertension with proteinuria by day 17 (early third trimester) [68]. Eremina et al. developed a tetracycline-inducible, podocyte-specific VEGF-knockout mouse using the Cre-loxP system. Upon stimulation with tetracycline these mice developed proteinuria with the characteristic lesions in glomeruli [69]. Bevacizumab is a humanized neutralizing monoclonal antibody against all human VEGF-A isoforms [70]. A systematic analysis of 7 trials showed that proteinuria developed in 20–60% and hypertension in 3–36% of Bevacizumab-treated patients [71]. Other VEGF-A antagonists have also been implicated [72]. Among reported patients, those with available histology are few and suggest variable preservation of podocyte foot processes. This could suggest a continuum of injury that begins in the endothelial cells of these patients with the loss or reduction of podocyte VEGF-A. The podocyte dedifferentiation observed in VEGF-null mice and the FPE encountered in some human biopsies may then imply the lack of a VEGF-dependent endothelial signal necessary for proper podocyte function.

Another example of a podocyte-secreted signal acting on endothelial cells is stromal cell-derived factor 1 (SDF-1 or CXCL12). Glomerular endothelial cells express CXCR4, the cognate receptor of SDF-1. Absence of either CXCR4 or SDF-1 gives rise to identical and lethal phenotypes in mice. In the developing nephron, SDF-1 producing podocytes and CXCR4-expressing endothelial cells appear to be aligned adjacent to each other. Further, glomeruli from endothelium-specific, CXCR4-knockout mice showed substantial endothelial cell detachment suggesting a key role for SDF-1/CXCR4 signalling in normal glomerulogenesis [73]. More recently, significant upregulation of CXCR4 transcripts was observed in cultured human microvascular endothelial cells in response to Shigella dysenteriae toxin-1 (STX), the causative agent in diarrhea-associated HUS. Children with documented *E. coli * O157:H7 infection who went on to develop HUS later had 4-fold higher SDF-1 levels than infected individuals who did not develop HUS. Furthermore, mice treated with SDF-1 inhibitor along with STX exposure, showed improved survival and abrogated HUS phenotype when compared to control mice [74]. This implies that the SDF-1/CXCR4 pathway is important in the development of diarrhea-associated HUS.

## 6. Mesangial Cell: Podocyte Crosstalk in Glomerular Disease

Mesangial cells are specialized pericytes whose primary functions are to provide structural support, regulate blood flow of the glomerular capillaries by their contractile activity, and control the turnover of mesangial matrix. Mesangial cells have been shown to synthesize transforming growth factor-*β* (TGF-*β*)—a key mediator in kidney disease progression. They express receptors for and respond to vasoactive substances including angiotensin-II, and many cytokines including TGF-*β* [75]. Experimentally, the central role of mesangial cells in glomerular development was implied by platelet-derived growth factor (PDGF) deficient or PDGF-*β* receptor deficient mice which lack mesangial cells and fail to develop glomerular tufts [76, 77]. Rats treated with rabbit antithymocyte serum sequentially develop severe complement-mediated mesangial cell death (with little involvement of other intraglomerular cell types) followed by mesangial proliferation, marked matrix expansion, and renal failure [78]. Further, proliferation of mesangial cells and expansion of mesangial matrix are hallmarks of many glomerular diseases [79]. 

Mesangial matrix expansion is typical in diabetic glomerulosclerosis suggesting a role for mesangial cells. In in vitro models of glycemic injury to mesangial cells, high-glucose media appear to increase synthesis of collagen I and IV. This effect appeared to be controlled in an autocrine fashion by mesangial TGF-*β* synthesis and was blocked by anti-TGF antibody [80]. Further, transfer of bone marrow (BM) cells from diabetic db/db mice into irradiated naïve B6 mice induced mesangial matrix expansion and albuminuria. Importantly these mice did not themselves develop diabetes or impaired glucose tolerance. The authors concluded that BM-derived mesangial cell progenitors were responsible for transmitting the diabetic nephropathy phenotype to naïve mice suggesting that mesangial cells exert a key influence in this disease [81]. 

The cross-talk between mesangial cells and podocytes culminating in proteinuria and progressive renal failure is best exemplified in IgA nephropathy. Polymeric hypo-galctosylated IgA molecules appear to be central to IgA nephropathy [82]. Polymeric IgA molecules from patients with IgA nephropathy have been shown to induce phenotypic, secretory and proliferative changes in mesangial cells [83]. TGF-*β*, tumor necrosis factor-*α* (TNF-*α*) and renin-angiotensin-aldosterone axis genes are upregulated in mesangial cells upon polymeric IgA treatment [83]. These IgA-molecules, however, are unable to directly induce podocyte changes in culture. Instead when medium from mesangial cells cultured in the presence of polymeric IgA is added to podocyte culture, it caused decreased expression of podocyte differentiation markers. Furthermore, similarly cultured podocytes showed markedly increased expression of TNF-*α* and TNF-*α* receptors 1 and 2 [84]. TNF-*α*, TGF-*β*, and angiotensin-II have been shown to be cytokines of central importance in the progression of interstitial fibrosis in IgA nephropathy [83, 85]. Together, these examples of mesangio-podocyte interaction could help devise a therapeutic strategy in these diseases centered around the mesangial cell. In summary, we have attempted to highlight the interdependence among the principal components of the glomerular filtration apparatus that is vital to its integrity. Injury to these individual components or disruption of intercomponent relationships seems to bring out both specific and common disease phenotypes often characterized by glomerular proteinuria. Better characterization of the key molecules involved in human diseases along with gene-targeting studies in experimental animals has considerably furthered our understanding of this crucial ongoing intraglomerular crosstalk and may ultimately make possible specific targeting of these pathways to mitigate and/or treat glomerular disease. 

## Figures and Tables

**Table 1a:** (a) Inherited

*Proteins involved in podocyte cytoskeletal integrity* [4]	
Podocin (AR), nephrin (AR), CD2-associated protein (AD), transient receptor potential cation-6 (AD), *α*-actinin-4 (AD), phospholipase-C*α*1 (AR), tetraspanin CD-151 (AR), and Wilm's tumor-1 (AD)	
*Basement membrane proteins* [9, 11]	
Alport's syndrome-*α*-4 collagen-5 (XL~85%); Alport's syndrome-*α*-4 collagen-3/4 (AR or AD~15%), thin Basement membrane disease (AD), and laminin-*β*2 (AR)	
*Proteins involved in endothelial/microvascular integrity* [12, 15]	
Complement regulatory proteins—complement factors H and I (AD), membrane cofactor protein (AD), and complement C3 (Gain-of-function)	
*Lysosomal storage diseases*—*metabolic injury* [9]	
Fabry's disease (XL)	

**Table 1b:** (b) Acquired

Immune	Nonimmune	Idiopathic
*Immune complex mediated* [13, 15]	*Hemodynamic injury* [10]	*Unclear mechanisms*
*In situ* formation or circulating complexes (postinfectious glomerulonephritis—streptococcal,	Hypertension	Minimal-change disease [22]
hepatits B/hepatitis-C-associated GN, systemic lupus erythematosus,	Adaptive hyperfiltration (nephron loss)	Membranous nephropathy [14]
and IgA nephropathy) idiopathic MP GN (types I and III)	*Other*	Primary FSGS [10]
*Antibody-mediated* [13]	Metabolic-hyperglycemia in diabetes	
Anti-GBM antibody disease	Deposition disease- Amyloidosis	
*T-cell-mediated injury* [13]	Toxic—Pamidronate, NSAIDs, D-pencillamine, and gold	
Antineutrophil cytoplasm antibody vasculitis, most glomerulonephritides	Infectious—HIV [10]	
*Complement-mediated injury* [12, 15]		
MPGN type II, atypical HUS		

Inheritance pattern—autosomal recessive (AR), dominant (AD), and X-linked (XL).

## References

[1] Peterson JC, Adler S, Burkart JM (1995). Blood pressure control, proteinuria, and the progression of renal disease: the modification of diet in renal disease study. *Annals of Internal Medicine*.

[2] Chronic Kidney Disease Prognosis C, Matsushita K, van der Velde M (2010). Association of estimated glomerular filtration rate and albuminuria with all-cause and cardiovascular mortality in general population cohorts: a collaborative meta-analysis. *The Lancet*.

[3] Carroll MF, Temte JL (2000). Proteinuria in adults: a diagnostic approach. *American Family Physician*.

[4] Barratt J, Topham P (2007). Urine proteomics: the present and future of measuring urinary protein components in disease. *Canadian Medical Association Journal*.

[5] Russo LM, Sandoval RM, McKee M (2007). The normal kidney filters nephrotic levels of albumin retrieved by proximal tubule cells: retrieval is disrupted in nephrotic states. *Kidney International*.

[6] Tanner GA (2009). Glomerular sieving coefficient of serum albumin in the rat: a two-photon microscopy study. *American Journal of Physiology*.

[7] Peti-Peterdi J (2009). Independent two-photon measurements of albumin GSC give low values. *American Journal of Physiology*.

[8] Tojo A, Endou H (1992). Intrarenal handling of proteins in rats using fractional micropuncture technique. *American Journal of Physiology*.

[9] Pavenstädt H, Kriz W, Kretzler M (2003). Cell biology of the glomerular podocyte. *Physiological Reviews*.

[10] D'Agati VD, Kaskel FJ, Falk RJ (2011). Focal segmental glomerulosclerosis. *The New England Journal of Medicine*.

[11] Hudson BG, Tryggvason K, Sundaramoorthy M, Neilson EG (2003). Alport’s syndrome, Goodpasture’s syndrome, and type IV collagen. *The New England Journal of Medicine*.

[12] Noris M, Remuzzi G (2009). Atypical hemolytic-uremic syndrome. *The New England Journal of Medicine*.

[13] Ambrus JL, Sridhar NR (1997). Immunologic aspects of renal disease. *Journal of the American Medical Association*.

[14] Kerjaschki D, Sharkey DJ, Farquhar MG (1984). Identification and characterization of podocalyxin—the major sialoprotein of the renal glomerular epithelial cell. *Journal of Cell Biology*.

[15] Schnabel E, Anderson JM, Farquhar MG (1990). The tight junction protein ZO-1 is concentrated along slit diaphragms of the glomerular epithelium. *Journal of Cell Biology*.

[16] Reiser J, Kriz W, Kretzler M, Mundel P (2000). The glomerular slit diaphragm is a modified adherens junction. *Journal of the American Society of Nephrology*.

[17] Ruotsalainen V, Ljungberg P, Wartiovaara J (1999). Nephrin is specifically located at the slit diaphragm of glomerular podocytes. *Proceedings of the National Academy of Sciences of the United States of America*.

[18] Wartiovaara J, Öfverstedt LG, Khoshnoodi J (2004). Nephrin strands contribute to a porous slit diaphragm scaffold as revealed by electron tomography. *Journal of Clinical Investigation*.

[19] Liu G, Kaw B, Kurfis J, Rahmanuddin S, Kanwar YS, Chugh SS (2003). Neph1 and nephrin interaction in the slit diaphragm is an important determinant of glomerular permeability. *Journal of Clinical Investigation*.

[20] Kaplan JM, Kim SH, North KN (2000). Mutations in ACTN4, encoding *α*-actinin-4, cause familial focal segmental glomerulosclerosis. *Nature Genetics*.

[21] Brown EJ, Schlöndorff JS, Becker DJ (2010). Mutations in the formin gene *INF2* cause focal segmental glomerulosclerosis. *Nature Genetics*.

[22] Ma H, Togawa A, Soda K (2010). Inhibition of podocyte FAK protects against proteinuria and foot process effacement. *Journal of the American Society of Nephrology*.

[23] Branten AJW, Van den Born J, Jansen JLJ, Assmann KJM, Wetzels JFM, Dijkman HBPM (2001). Familial nephropathy differing from minimal change nephropathy and focal glomerulosclerosis. *Kidney International*.

[24] Macconi D, Ghilardi M, Bonassi ME (2000). Effect of angiotensin-converting enzyme inhibition on glomerular basement membrane permeability and distribution of zonula occludens-1 in MWF rats. *Journal of the American Society of Nephrology*.

[25] Karumanchi SA, Maynard SE, Stillman IE, Epstein FH, Sukhatme VP (2005). Preeclampsia: a renal perspective. *Kidney International*.

[26] Wharram BL, Goyal M, Gillespie PJ (2000). Altered podocyte structure in GLEPP1 (Ptpro)-deficient mice associated with hypertension and low glomerular filtration rate. *Journal of Clinical Investigation*.

[27] van den Berg JG, van den Bergh Weerman MA, Assmann KJM, Weening JJ, Florquin S (2004). Podocyte foot process effacement is not correlated with the level of proteinuria in human glomerulopathies. *Kidney International*.

[28] Seiler MW, Venkatachalam MA, Cotran RS (1975). Glomerular epithelium: structural alterations induced by polycations. *Science*.

[29] Garg P, Verma R, Cook L (2010). Actin-depolymerizing factor cofilin-1 is necessary in maintaining mature podocyte architecture. *Journal of Biological Chemistry*.

[30] Kim JH, Wu H, Green G (2003). CD2-associated protein haploinsufficiency is linked to glomerular disease susceptibility. *Science*.

[31] Wei C, Reiser J (2011). Minimal change disease as a modifiable podocyte paracrine disorder. *Nephrology Dialysis Transplantation*.

[32] Garg P, Rabelink T (2011). Glomerular proteinuria: a complex interplay between unique players. *Advances in Chronic Kidney Disease*.

[33] Reiser J, Oh J, Shirato I (2004). Podocyte migration during nephrotic syndrome requires a coordinated interplay between cathepsin L and *α*3 integrin. *Journal of Biological Chemistry*.

[34] Adler S, Chen X (1992). Anti-Fx1A antibody recognizes a *β*1-integrin on glomerular epithelial cells and inhibits adhesion and growth. *American Journal of Physiology*.

[35] El-Aouni C, Herbach N, Blattner SM (2006). Podocyte-specific deletion of integrin-linked kinase results in severe glomerular basement membrane alterations and progressive glomerulosclerosis. *Journal of the American Society of Nephrology*.

[36] Kang YS, Li Y, Dai C, Kiss LP, Wu C, Liu Y (2010). Inhibition of integrin-linked kinase blocks podocyte epithelial-mesenchymal transition and ameliorates proteinuria. *Kidney International*.

[37] Blasi F, Carmeliet P (2002). uPAR: a versatile signalling orchestrator. *Nature Reviews Molecular Cell Biology*.

[38] Smith HW, Marshall CJ (2010). Regulation of cell signalling by uPAR. *Nature Reviews Molecular Cell Biology*.

[39] Wei C, Moller CC, Altintas MM, " (2008). Modification of kidney barrier function by the urokinase receptor. *Nature Medicine*.

[40] Wei C, El Hindi S, Li J (2011). Circulating urokinase receptor as a cause of focal segmental glomerulosclerosis. *Nature Medicine*.

[41] Striker GE, Smuckler EA (1970). An ultrastructural study of glomerular basement membrane synthesis. *American Journal of Pathology*.

[42] Abrahamson DR (1987). Structure and development of the glomerular capillary wall and basement membrane. *American Journal of Physiology*.

[43] Sanes JR, Engvall E, Butkowski R, Hunter DD (1990). Molecular heterogeneity of basal laminae: isoforms of laminin and collagen IV at the neuromuscular junction and elsewhere. *Journal of Cell Biology*.

[44] Kalluri R, Cosgrove D (2000). Assembly of Type IV collagen. Insights from *α*3(IV) collagen-deficient mice. *Journal of Biological Chemistry*.

[45] Zenker M, Aigner T, Wendler O (2004). Human laminin *β*2 deficiency causes congenital nephrosis with mesangial sclerosis and distinct eye abnormalities. *Human Molecular Genetics*.

[46] Noakes PG, Miner JH, Gautam M, Cunningham JM, Sanes JR, Merlie JP (1995). The renal glomerulus of mice lacking S-laminin/laminin *β*2: nephrosis despite molecular compensation by laminin *β*1. *Nature Genetics*.

[47] Reeves WH, Kanwar YS, Gist Farquhar M (1980). Assembly of the glomerular filtration surface. Differentiation of anionic sites in glomerular capillaries of newborn rat kidney. *Journal of Cell Biology*.

[48] Kanwar YS, Farquhar MG (1979). Anionic sites in the glomerular basement membrane. In vivo and vitro localization to the laminae rarae by cationic probes. *Journal of Cell Biology*.

[49] Kanwar YS, Linker A, Farquhar MG (1980). Increased permeability of the glomerular basement membrane to ferritin after removal of glycosaminoglycans (heparan sulfate) by enzyme digestion. *Journal of Cell Biology*.

[50] Bolton GR, Deen WM, Daniels BS (1998). Assessment of the charge selectivity of glomerular basement membrane using Ficoll sulfate. *American Journal of Physiology*.

[51] Daniels BS (1994). Increased albumin permeability in vitro following alterations of glomerular charge is mediated by the cells of the filtration barrier. *Journal of Laboratory and Clinical Medicine*.

[52] Guasch A, Deen WM, Myers BD (1993). Charge selectivity of the glomerular filtration barrier in healthy and nephrotic humans. *Journal of Clinical Investigation*.

[53] Chen S, Wassenhove-McCarthy DJ, Yamaguchi Y (2008). Loss of heparan sulfate glycosaminoglycan assembly in podocytes does not lead to proteinuria. *Kidney International*.

[54] Clement LC, Avila-Casado C, MacÉ C (2011). Podocyte-secreted angiopoietin-like-4 mediates proteinuria in glucocorticoid-sensitive nephrotic syndrome. *Nature Medicine*.

[55] Ballermann BJ (2007). Contribution of the endothelium to the glomerular permselectivity barrier in health and disease. *Nephron*.

[56] Jeansson M, Haraldsson B (2003). Glomerular size and charge selectivity in the mouse after exposure to glucosaminoglycan-degrading enzymes. *Journal of the American Society of Nephrology*.

[57] Hjalmarsson C, Johansson BR, Haraldsson B (2004). Electron microscopic evaluation of the endothelial surface layer of glomerular capillaries. *Microvascular Research*.

[58] Fridén V, Oveland E, Tenstad O (2011). The glomerular endothelial cell coat is essential for glomerular filtration. *Kidney International*.

[59] Kerr H, Richards A (2012). Complement-mediated injury and protection of endothelium: lessons from atypical haemolytic uraemic syndrome. *Immunobiology*.

[60] Besbas N, Karpman D, Landau D (2006). A classification of hemolytic uremic syndrome and thrombotic thrombocytopenic purpura and related disorders. *Kidney International*.

[61] Manuelian T, Hellwage J, Meri S (2003). Mutations in factor H reduce binding affinity to C3b and heparin and surface attachment to endothelial cells in hemolytic uremic syndrome. *Journal of Clinical Investigation*.

[62] Heinen S, Józsi M, Hartmann A (2007). Hemolytic uremic syndrome: a factor H mutation (E1172Stop) causes defective complement control at the surface of endothelial cells. *Journal of the American Society of Nephrology*.

[63] Richards A, Kemp EJ, Liszewski MK (2003). Mutations in human complement regulator, membrane cofactor protein (CD46), predispose to development of familial hemolytic uremic syndrome. *Proceedings of the National Academy of Sciences of the United States of America*.

[64] Foster RR, Hole R, Anderson K (2003). Functional evidence that vascular endothelial growth factor may act as an autocrine factor on human podocytes. *American Journal of Physiology*.

[65] Eremina V, Quaggin SE (2004). The role of VEGF-A in glomerular development and function. *Current Opinion in Nephrology and Hypertension*.

[66] Sison K, Eremina V, Baelde H (2010). Glomerular structure and function require paracrine, not autocrine, VEGF-VEGFR-2 signaling. *Journal of the American Society of Nephrology*.

[67] Eremina V, Sood M, Haigh J (2003). Glomerular-specific alterations of VEGF-A expression lead to distinct congenital and acquired renal diseases. *Journal of Clinical Investigation*.

[68] Maynard SE, Min JY, Merchan J (2003). Excess placental soluble fms-like tyrosine kinase 1 (sFlt1) may contribute to endothelial dysfunction hypertension, and proteinuria in preeclampsia. *Journal of Clinical Investigation*.

[69] Eremina V, Jefferson JA, Kowalewska J (2008). VEGF inhibition and renal thrombotic microangiopathy. *The New England Journal of Medicine*.

[70] Presta LG, Chen H, O’Connor SJ (1997). Humanization of an anti-vascular endothelial growth factor monoclonal antibody for the therapy of solid tumors and other disorders. *Cancer Research*.

[71] Zhu X, Wu S, Dahut WL, Parikh CR (2007). Risks of proteinuria and hypertension with bevacizumab, an antibody against vascular endothelial growth factor: systematic review and meta-analysis. *American Journal of Kidney Diseases*.

[72] Izzedine H, Brocheriou I, Deray G, Rixe O (2007). Thrombotic microangiopathy and anti-VEGF agents. *Nephrology Dialysis Transplantation*.

[73] Takabatake Y, Sugiyama T, Kohara H (2009). The CXCL12 (SDF-1)/CXCR4 axis is essential for the development of renal vasculature. *Journal of the American Society of Nephrology*.

[74] Petruzziello-Pellegrini TN, Yuen DA, Page AV (2012). The CXCR4/CXCR7/SDF-1 pathway contributes to the pathogenesis of Shiga toxin-associated hemolytic uremic syndrome in humans and mice. *Journal of Clinical Investigation*.

[75] Schlöndorff D (1996). Roles of the mesangium in glomerular function. *Kidney International*.

[76] Leveen P, Pekny M, Gebre-Medhin S, Swolin B, Larsson E, Betsholtz C (1994). Mice deficient for PDGF B show renal, cardiovascular, and hematological abnormalities. *Genes and Development*.

[77] Soriano P (1994). Abnormal kidney development and hematological disorders in PDGF *β*- receptor mutant mice. *Genes and Development*.

[78] Yamamoto T, Mundy CA, Wilson CB, Blantz RC (1991). Effect of mesangial cell lysis and proliferation on glomerular hemodynamics in the rat. *Kidney International*.

[79] Buschhausen L, Seibold S, Gross O, Matthaeus T, Weber M, Schulze-Lohoff E (2001). Regulation of mesangial cell function by vasodilatory signaling molecules. *Cardiovascular Research*.

[80] Ziyadeh FN, Sharma K, Ericksen M, Wolf G (1994). Stimulation of collagen gene expression and protein synthesis in murine mesangial cells by high glucose is mediated by autocrine activation of transforming growth factor-*β*. *Journal of Clinical Investigation*.

[81] Zheng F, Cornacchia F, Schulman I (2004). Development of albuminuria and glomerular lesions in normoglycemic B6 recipients of db/db mice bone marrow: the role of mesangial cell progenitors. *Diabetes*.

[82] Tomana M, Novak J, Julian BA, Matousovic K, Konecny K, Mestecky J (1999). Circulating immune complexes in IgA nephropathy consist of IgA1 with galactose-deficient hinge region and antiglycan antibodies. *Journal of Clinical Investigation*.

[83] Lai KN, Tang SCW, Guh JY (2003). Polymeric IgA1 from patients with IgA nephropathy upregulates transforming growth factor-*β* synthesis and signal transduction in human mesangial cells via the renin-angiotensin system. *Journal of the American Society of Nephrology*.

[84] Lai KN, Leung JCK, Chan LYY (2008). Activation of podocytes by mesangial-derived TNF-*α*: glomerulo-podocytic communication in IgA nephropathy. *American Journal of Physiology*.

[85] Del Prete D, Gambaro G, Lupo A (2003). Precocious activation of genes of the renin-angiotensin system and the fibrogenic cascade in IgA glomerulonephritis. *Kidney International*.

